# Intracerebral Hemorrhage: Blood Components and Neurotoxicity

**DOI:** 10.3390/brainsci9110316

**Published:** 2019-11-09

**Authors:** Neha Madangarli, Frederick Bonsack, Rajaneekar Dasari, Sangeetha Sukumari–Ramesh

**Affiliations:** Department of Pharmacology and Toxicology, Medical College of Georgia, Augusta University, 1120 15th Street, Augusta, GA 30912, USA; nmadangarli@augusta.edu (N.M.); fbonsack@augusta.edu (F.B.);

**Keywords:** ICH, hemin, thrombin, hemoglobin, iron

## Abstract

Intracerebral hemorrhage (ICH) is a subtype of stroke which is associated with the highest mortality and morbidity rates of all strokes. Although it is a major public health problem, there is no effective treatment for ICH. As a consequence of ICH, various blood components accumulate in the brain parenchyma and are responsible for much of the secondary brain damage and ICH-induced neurological deficits. Therefore, the strategies that could attenuate the blood component-induced neurotoxicity and improve hematoma resolution are highly needed. The present article provides an overview of blood-induced brain injury after ICH and emphasizes the need to conduct further studies elucidating the mechanisms of hematoma resolution after ICH.

## 1. Introduction 

Intracerebral Hemorrhage (ICH) is a devastating sub-type of stroke caused by blood vessel rupture in the brain and the subsequent bleeding into the surrounding tissue [1]. ICH is the second most common form of stroke following ischemic stroke, and it accounts for 10–20% of all strokes worldwide. The prevalence of ICH varies across countries, ethnicities, and race. For instance, in Asian and Western countries, ICH accounts for 18–24% and 8–15% of all stroke cases, respectively [2], and the incidence of ICH in black Americans is twice as high as in whites [3,4]. Further, its occurrence is higher in countries of low and middle income, and those ICH cases often result in fatality [5], which could partly be due to the reduced or lack of access to health care. 

ICH is associated with the highest mortality and morbidity rates of all strokes. The mortality rate of ICH at one month is approximately 40%, which has not changed over the past twenty years [6]. Furthermore, if the patient survives the ictus, then the resulting hematoma within brain parenchyma triggers a series of adverse events causing secondary insults and long term neurological deficits [7,8]. Consistently, ICH is one of the leading causes of long term morbidity, and 74% of the survivors remain functionally dependent 12 months after the ictus [6,9]. Importantly, ICH imposes a significant economic burden on, society contributing to an estimated $17.2 billion in annual direct costs to the US healthcare system associated with stroke [10,11,12,13]. ICH is more than twice as common as subarachnoid hemorrhage (SAH) [14], and results in more disability than SAH [15]. Though there is a substantial difference in the incidence rate of ischemic and hemorrhagic stroke, a recent report attributed 3.2 million deaths to ICH, while 3.3 million deaths were attributed to ischemic stroke in the year 2013, further highlighting devastating nature of ICH [16]. 

Advanced age is regarded as a risk factor of ICH, [6] and ICH incidence increases exponentially with increase in age [14]. Consistently, the annual incidence rate of ICH per 100,000 individuals increased from 5.9 in younger (35–54 year old) to 176.3 in older (75–94 year old) age groups [2]. Further, age is strongly associated with outcome after ICH [17]. Along these lines, an unfavorable outcome (modified Rankin Scale score >2) was found more often in older patients compared to younger ones [18]. Importantly, the worldwide incidence of ICH has risen by ~47% over the last 20 years [2], and hospital admissions have increased by 18% in the past ten years [19]. This could be due to the increase in the number of older adults. Though the gender differences in outcome have not been fully characterized in the pathophysiology of ICH, it is reported that age and gender interact to affect patient outcome after ICH. For younger patients, female sex was protective; however, at ages higher than 60 years, female sex was a risk factor for discharge to hospice or death [20]. In addition, females experienced more a right hemispheric ICH than males, which could also influence functional recovery and mortality outcomes [21]. However, no significant difference was found between males and females in the incidence rate of ICH [6]. 

Hypertension is considered as a major risk factor of ICH [22], and usually it leads to rupture of vessels at the bifurcation of small arteries within the brain [1]. Hypertension increases the risk of ICH approximately two-fold [23] and is associated with 83% of ICH patients [24]. Another risk factor that accounts for ~20% of ICH is cerebral amyloid angiopathy, which is characterized by the deposition of amyloid-β plaques in capillaries, arterioles, and small arteries within the brain. Cerebral amyloid angiopathy often results in sporadic intracerebral hemorrhage in elderly people. In addition, white-matter abnormalities seem to increase the risk of both sporadic and familial intracerebral hemorrhage, implicating a prominent role of vascular deformities in the onset of ICH. Age increases the risk of comorbidities, including hypertension, vascular deformities, and cerebral amyloid angiopathy, that contribute to the pathology of ICH [2,22]. Though chronic hypertension, amyloid angiopathy, and advanced age are recognized as the prominent risk factors of ICH, other risk factors also include, but are not limited to, smoking, diabetes, drug abuse, use of anticoagulants, and alcohol intake [25]. As the population ages, the incidence of ICH, due to amyloid angiopathy, may further rise [22], and the incidence is expected to have doubled by 2050 [26] due to aging and the spreading use of anticoagulants [27]. 

Despite an overall increase in preclinical studies, the pathophysiology of ICH remains largely enigmatic, which is partly responsible for lack of treatment options. The present article provides an overview of blood-induced brain injury after ICH and emphasizes the need to conduct further studies, elucidating the mechanisms of hematoma resolution after ICH. The data pertinent to the present article was collected by the PubMed database search with no time limitation and using the search terms intracerebral hemorrhage, thrombin, hemoglobin, hemin, and iron.

## 2. Primary and Secondary Brain Injury 

ICH results in both primary and secondary brain damage. The primary brain damage is mainly attributed to the mass effect of hematoma, whereas the extravasated blood components induce inflammatory and oxidative stress pathways contributing to secondary brain damage. The hematoma is not a static entity; it is highly dynamic in nature due to bleeding and rebleeding and it disrupts the surrounding brain structures, resulting in early neurological dysfunction. Secondary brain injury evolves over a period of time (from hours to days) after the primary brain injury, and it includes an entire cascade of cellular and molecular changes in the brain that contribute to further destruction of brain tissue. Though the secondary brain injury often leads to severe neurological deficits and sometimes delayed fatality [28,29], a detailed mechanistic understanding of the detrimental events underlying secondary injury after ICH is lacking [26,30,31,32,33]. In addition, several promising clinical trials have failed to demonstrate patient benefits after ICH. Along these lines, the optimal therapy targeting ICH-induced primary brain injury has not yielded conclusive benefits in clinical trials thus far [34]. Therefore, research on the mechanisms underlying ICH-induced secondary brain injury in search for novel therapeutic targets is warranted. Importantly, many patients continue to deteriorate despite no signs of hematoma expansion, and there is increasing interest in the mechanisms of secondary brain injury following ICH. The key factors that contribute to secondary brain injury after ICH are thrombin, hemoglobin, hemin, and iron—the blood components that are known to activate cytotoxic, excitotoxic, oxidative, and inflammatory pathways [7].

### 2.1. Thrombin and ICH

Thrombin is a trypsin-like allosteric serine protease essential to blood coagulation, and it gets produced on the plasma membranes of platelets, neutrophils, monocytes, and lymphocytes as a result of cleavage of its inactive precursor, prothrombin, following activation of the blood coagulation cascade [35]. Cerebral hemorrhage activates coagulation cascade, which in turn results in the release and subsequent brain accumulation of large amounts of thrombin. Thrombin modulates many intracellular signaling pathways [36] such as mitogen-activated protein kinase (MAPK) and phosphoinositide 3-kinase (PI3K) signaling in the brain [37,38], which play roles in glial activation, neuronal survival, and neurogenesis. Importantly, intracerebral infusion of thrombin in rodents resulted in the up-regulation of proinflammatory cytokines, disruption of the blood–brain barrier (BBB), neurotoxicity, DNA fragmentation, activation of Src kinase, modulation of N-methyl-D-aspartate (NMDA) receptor function, augmented expression of matrix metalloproteinase-*9* (MMP-9), an increase of brain edema, and neurological deficits implicating the neurotoxic potential of thrombin [39,40,41,42,43,44,45,46,47]. Thrombin also plays a critical role in inducing water channels such as aquaporin-4 (AQP-4) and aquaporin-9 (AQP-9) that contribute to cerebral edema development after ICH [48]. The thrombin inhibitor hirudin attenuated blood-induced cerebral edema in rats [49]. Further, thrombin brain infusions produced focal motor seizures in rats [50]. Of note, thrombin-induced brain injury occurs mainly via the G-protein-coupled receptor, PAR (protease-activated receptor). Protease-activated receptor-1 (PAR-1), a subtype of the PAR receptor, is found in neurons, oligodendrocytes, and glial cells, and the activation of PAR potentiates NMDA receptor responses [42] and modulates glial response to a brain injury [51]. As thrombin is capable of activating glial cells, it is also regarded as a proinflammatory agent [52,53]. In microglia p38 mitogen-activated protein kinase (p38 MAPK), c-Jun N-terminal kinases (JNK) and NACHT, LRR, and PYD domains containing protein 3 (NLRP3) inflammasome are activated by thrombin and thrombin-induced microglial activation involves PAR subtypes, PAR-1, and PAR-4 [52,53,54]. In addition, thrombin induces direct neurotoxicity at nanomolar to micromolar concentrations. To this end, 10 nM–10 µM of thrombin induced neuronal death. In contrast, 10 pM–10 nM of thrombin protected hippocampal neurons against various cellular insults [51,55]. Further, consistent with the neuroprotective role of thrombin at low concentrations, it is reported that preconditioning with a low dose of thrombin attenuated brain edema after ICH [56]. Furthermore, thrombin could augment neurogenesis after ICH [57]. However, the precise functional role of thrombin in neuroprotection, neurogenesis, and thereby brain recovery is yet to be defined. 

### 2.2. Hemoglobin and ICH

As a consequence of red blood cell (RBC) lysis following intracerebral hemorrhage (ICH), hemoglobin (Hb) is released into the extracellular space. A hemoglobin molecule contains four heme groups and a globin, and each heme group consists of a porphyrin ring with ferrous iron at the center. Upon release, the iron in the Hb subunit gets oxidized from ferrous (2+) to ferric (3+). This destabilizes Hb molecules [58] and triggers a cascade of inflammatory reactions leading to blood–brain barrier disruption, development of peri-hematomal edema, neuronal death, and secondary brain damage after brain hemorrhage [59]. The presence of free Hb in brain tissue is suggested to exacerbate oxidative [60] as well as inflammatory brain damage [61]. To this end, intracerebral infusion of hemoglobin causes an increase in brain water content [61]. In addition, as Hb is one of the major components of blood, it is suggested to play a crucial role in ICH-induced neuronal damage [62,63]. Therefore, the timely clearance of Hb after ICH is critical. 

One of the endogenous receptors responsible for the clearance of Hb is CD163 [64]. The cysteine-rich scavenger receptor CD163 binds to and facilitates the endocytosis and subsequent clearance of Hb that is bound to the plasma glycoprotein haptoglobin (Hp) [64]. The formation of the Hb–Hp complex also protects Hb from oxidative modifications [65]. Along these lines, in the Hb–Hp complex, the iron moiety is sequestered within the hydrophobic pocket of Hb, blocking its oxidative and cytotoxic activities [55]. In a physiological condition, haptoglobin levels are low in the brain, but the expression of haptoglobin increases after ICH, and it can also enter the brain through circulation after a brain injury [66]. Of note, overexpression of haptoglobin alleviates brain injury after experimental ICH [66]. Furthermore, patients with naturally high levels of macrophage/microglial CD163 may have faster rates of hematoma resorption, and/or less neuroinflammation due to rapid sequestration of toxic hemoglobin [67]. Further, CD163 expression increases over time in the brain after ICH [4]. In human post mortem brains and in a porcine ICH model, activated microglia/macrophages surrounding the hematoma express CD163 [4,68,69], implicating a role of microglia/macrophages in Hb clearance after ICH. Altogether, Hp–Hb–CD163 acts as the main pathway in Hb scavenging, and thereby exerts a neuroprotective role [69]. Further, reduction in hemoglobin toxicity attenuated brain injury, and improved functional outcomes in preclinical models of ICH [61,66]. However, recent studies demonstrated a differential role of CD163 [70]. Along these lines, CD163 exerted a neurotoxic effect in the acute stage while it was neuroprotective in the subacute phase after ICH, warranting further investigation. 

Apart from microglial or macrophages, CD163 is also up-regulated acutely in neurons following ICH [71,72]. Along these lines, Hb released after intraventricular hemorrhage is taken up into neurons via CD163, causing increased cellular iron leading to neuronal death [73,74]. Further, CD163 has been shown to detach from the cell surface, resulting in a soluble form that circulates in the plasma of ICH patients [75]. Acute serum-soluble CD163 (sCD163) levels are significantly associated with the expansion of hematoma volume and peri-hematomal edema [75]. Studies suggest that sCD163 also binds haptoglobin–hemoglobin complexes and plays a role in Hb clearance [76]. However, functional studies are required to determine the precise role of sCD163 in the pathophysiology of ICH. 

### 2.3. Hemin and ICH

Hemin, the degradation product of Hb, plays a critical role in ICH-associated inflammatory brain damage by activating microglia [77]. Along these lines, Toll-like receptor-4 (TLR4) is a key regulator of heme-mediated inflammatory brain damage after ICH [77] and intracerebral infusion of hemin in rodents resulted in elevated brain levels of IL-1β and cerebral edema [61,78]. Further, heme activates Toll-like receptor-2 (TLR-2), cofilin, and NLRP3 signaling, contributing to inflammatory brain damage after ICH [79,80,81]. Hemin reacts with peroxides to produce highly reactive free radicals and is a pro-oxidant [82]. Furthermore, being lipophilic, hemin intercalates into the plasma membrane of cells, resulting in lipid peroxidation and membrane dysfunction [83]. Of note, hemin is toxic to neurons [84], resting glia, and microvascular cells [85] and causes cell death. However, it is reported that activated microglia is less susceptible to hemin-mediated cell death in comparison to resting microglia [86]. Free heme can also induce adhesion molecule expression and cause vascular permeability and leukocyte infiltration [84]. Further, hemin inhibits the Ca^2+^-regulated potassium channel in the brain mitochondria mitoBK_Ca_, leading to neurotoxicity [87]. Altogether, hemin plays a key role in secondary neuronal injury after ICH and hemin-mediated neurotoxicity is partly due to iron that is liberated as a consequence of hemin catabolism. Therefore, timely removal of hemin is essential for brain recovery and repair following ICH. To this end, the cells that are responsible for the sequestration and subsequent degradation of hemin are primarily glial cells. A 60 kDa serum glycoprotein, hemopexin, is responsible for the uptake of hemin into the brain cells via low-density lipoprotein receptor-related protein (LRP), expressed on astrocytes and neurons [88]. However, the concentration of hemin after ICH exceeds hemopexin levels in the serum, resulting in the accumulation of hemin in the extracellular space and that elicits neurotoxicity. Apart from hemopexin, heme carrier protein 1 (HCP1) also facilitates the endocytosis of hemin into astrocytes [89]. Within the cells, heme oxygenases (HO) are enzymes that are primarily responsible for the degradation of heme, and it occurs mainly via two isoenzymes, heme oxygenase-1 (HO-1) and heme oxygenase-2 (HO-2). 

HO-1, encoded for by the *HMOX 1* gene, is an inducible 32-kDa protein, which is up-regulated by stimuli such as heme, nitric oxide, heavy metals, growth factor, cytokines, and modified lipids [90,91,92,93]. HO-1 catalyzes the degradation of heme into equimolar amounts of iron, biliverdin, and CO, which plays critical roles in inflammation, oxidative stress, apoptosis, cell proliferation, fibrosis, and angiogenesis [94,95]. Though iron, catabolically derived from heme, elicits oxidative brain damage, the induction of HO-1 is often accompanied by the up-regulation of ferritin, a protective enzyme that can sequester iron. However, ferritin up-regulation after ICH could be insufficient to handle the iron that releases, leading to neurotoxicity. Upon release, biliverdin gets converted to bilirubin through the action of biliverdin reductase [96]. Further, bilirubin is toxic to the brain [97], although it has antioxidant properties at low concentrations [98,99]. Apart from immunomodulatory potential, a recent study demonstrated a key role of CO in erythrophagocytosis and subsequent clearance of hematoma after subarachnoid hemorrhage [100]. However, pharmacological inhibition of HO-1 reduced Hb-induced edema [61], improved neuroprotection [101], and attenuated both hematoma volume and cerebral edema after ICH [102]. Consistently, HO-1 activator exacerbated brain injury after ICH. Further, HO-1 knock out mice exhibited improved neurological outcomes and a reduction in the number of ionized calcium binding adaptor molecule 1(Iba1)-positive cells on day 1 post-ICH. Altogether the data implicates a neurotoxic role of HO-1 in the acute phase of ICH. Of note, recent studies demonstrated a neuroprotective role of HO-1 in the later stage of ICH. To this end, pharmacological activation of HO-1 during the later stages of ICH was associated with increased hematoma absorption, angiogenesis, and improved neurological function [103]. Though the cells that mainly express HO-1 after ICH in the acute phase are Iba1-positive microglia or macrophages [104], the precise functional role of HO-1 in microglia or macrophages after ICH remains largely unknown, and it requires investigation employing transgenic animal models. In the subacute and later phases of ICH, HO-1 expression is also observed in glial fibrillary acidic protein (GFAP)-positive astrocytes [104]. Furthermore, mice overexpressing HO-1 in GFAP-positive cells exhibited reduced brain injury in comparison to wild types, implicating a protective role of HO-1 in astrocytes [105]. However, the functional role of HO-1 in modulating long-term neurological outcomes after ICH requires further studies. 

Under normal conditions, HO-1 is barely detectable in the brain, and most of the heme oxygenase activity in the brain is from the HO-2 isoform. In contrast to HO-1, which contains multiple regulatory elements in its promoter region, the only functional response element in the promoter region of HO-2 is the glucocorticoid response element [106]. In addition, though HO-1 and HO-2 demonstrate alpha-helical secondary structures, the two isoenzymes differ in their C-terminal residues which, in turn, also play a regulatory role in the transcription of the enzymes. HO-1 expression is mostly increased in glial cells after a brain injury, whereas HO-2 is constitutively expressed in neurons [107,108]. HO-2 is expressed abundantly throughout the brain, where it is found to be localized in the forebrain, hippocampus, midbrain, basal ganglia, thalamic regions, cerebellum, and brain stem [109]. It has also been suggested that HO-2 is responsible for CO production in neuronal cell populations for homeostatic functions [109]. Furthermore, basal levels of HO-2 are involved in cellular defense mechanisms through the regulation of extracellular superoxide dismutase, Akt (protein kinase B), and apoptotic signaling kinase-1 (ASK-1) which, in turn, regulate the rate of heme degradation [110]. Further, HO-2^−/−^ mice showed more severe neurologic deficits than wild type mice acutely after ICH implicating a neuroprotective role of HO-2.

### 2.4. Iron and ICH

Iron is a major contributor to oxidative stress and secondary brain damage after ICH. Iron release into the brain tissue begins 24 h after hemorrhage and gets deposited in the perihematomal brain tissue. Free iron has the potential to generate highly cytotoxic hydroxyl radicals via the Fenton reaction (Fe(II) + H_2_O_2_ → Fe(III) +OH^−^ +^•^OH) leading to lipid peroxidation [111], and injection of ferrous iron into the cerebral cortices of rats causes lipid peroxidation within 15 min [112]. Further, hydroxyl radicals degrade lipid peroxides, which results in the production of hydroxyl, alkoxy, and peroxy radicals, causing further damage [66,113]. These radical-induced injuries to lipids, DNA, and proteins result in the death of neurons, glia, and endothelial cells. The injury of endothelial cells and subsequently the neurovascular unit results in blood–brain barrier (BBB) disruption and vasogenic edema [114]. ICH induces several types of cell death such as necrosis, apoptosis, necroptosis, autophagy, and ferroptosis [78,115,116,117,118]. Notably, pharmacological inhibition of ferroptosis, a recently characterized iron-induced non-apoptotic cell death, improved neurological outcome after ICH [119]. Importantly, iron has been established as an independent factor contributing to brain edema formation after ICH [120]. Iron is also responsible for the oxidative injury mediated by hemoglobin [84]. Furthermore, the administration of deferoxamine, a chelator of iron attenuated perihematomal iron accumulation, neurodegeneration, microglial activation, and white-matter injury [111,121] and improved neurological outcomes in pre-clinical models of ICH [122]. Systemic administration of minocycline, an iron chelator, attenuated ICH-induced brain overload of iron, brain damage, and neurological deficits in rats [123]. Divalent metal transporter1 (DMT1) and Ferroportin 1 (FPN1) are positively influenced by ferrous iron status in the brain after ICH, and both DMT1 and FPN1 attenuated iron overload after ICH by enhancing transmembrane iron export [124]. Importantly, brain accumulation of iron after ICH takes longer to be cleared and is partly responsible for delayed neurodegeneration, brain atrophy, and long term neurological deficits observed after ICH [125,126]. 

## 3. Hematoma Resolution and ICH 

**Figure 1:**
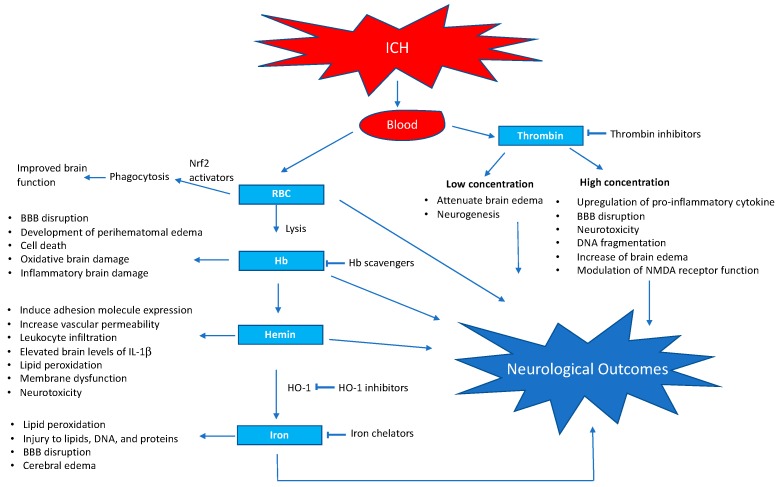
Schematic representation of blood-induced neurotoxicity after intra cerebral hemorrhage and selected points of action of pharmacological interventions. BBB, blood–brain barrier; NMDA, N-methyl-D-aspartate.

One of the key predictors of poor patient outcome after ICH is hematoma volume [127]. In clinical practice, neurological outcome is positively associated with the rate of hematoma absorption/resolution [6]. A larger hematoma may cause enhanced brain injury not only because of mass effect, but also because it results in the accumulation of cytotoxic blood components. This cytotoxic insult has complex oxidative and inflammatory components, ultimately leading to neurological dysfunction (Figure 1). Therefore, strategies to efficiently remove intra parenchymal blood may attenuate brain damage and improve functional recovery after ICH. In this regard, one of the transcription factors that is being explored in pre-clinical studies is nuclear factor erythroid 2-related factor 2 (Nrf2). Nrf2 cell signaling has been demonstrated to contribute to the regulation of a wide variety of antioxidant, detoxification, and cell survival genes [128]. Nrf2 activators increased HO-1 expression in astrocytes, and that reduced the vulnerability of astrocytes to hemin [129]. Several studies have documented the efficacy of Nrf2 activators in enhancing hematoma resolution, as well as attenuating oxidative and inflammatory brain damage, after ICH [130]. Further, hematoma resolution was also significantly impaired in Nrf2 knockout mice in comparison to controls, further confirming that Nrf2 plays a crucial role in hematoma resolution [131]. However, improved hematoma resolution observed with Nrf2 activators could also be due to the attenuation of inflammatory and oxidative signaling and subsequent prevention of hematoma expansion or improvement in brain recovery mechanisms. However, Nrf2 activators augmented the RBC phagocytosis by cultured microglia [130,132], and Nrf2 is a key regulator of CD36, a scavenger protein, which plays a role in microglial phagocytosis of RBC [132], implicating a critical role of Nrf2 in hematoma clearance. Importantly, one of the disadvantages of translating preclinical studies into a clinical trial is the reduced bioavailability of Nrf2 activators in humans [133,134]. Furthermore, many pharmacological Nrf2 activators activate Nrf2 by enhancing cellular stress [120,135,136] and are reactive electrophiles [136,137,138,139], which brings into question their clinical applicability. Further, the prolonged administration of an Nrf2 activator, dimethyl fumarate, causes lymphopenia in multiple sclerosis patients and advanced age increases the risk of lymphopenia [120,140]. Altogether, non-electrophilic therapeutic agents that can augment Nrf2 with increased bioavailability are highly needed to test the efficacy of targeting Nrf2 and thereby improving patient outcomes after ICH. Furthermore, though Nrf2 signaling plays a critical role after ICH, the precise molecular mechanisms involved in the activation of Nrf2 after ICH is not fully understood and it needs investigation. 

## 4. Conclusions and Future Directions

Overall, blood components play a critical role in inducing neurotoxicity and secondary brain damage after ICH and hence molecular mediators that can augment the removal of blood components are critical for improving neurological outcomes after ICH. Though the neurotoxic potential of high concentrations of thrombin could outweigh the neuroprotective effects of low concentrations of thrombin, future studies are warranted in elucidating the precise molecular mechanism by which low concentrations of thrombin confer neuroprotection after ICH. Furthermore, the precise functional role of CD163, as well as HO-1, in modulating long-term neurological outcomes after ICH warrants further study. Further, given the emerging role of Nrf2 in hematoma resolution after ICH, the intrinsic molecular regulators of Nrf2 after ICH require investigation. Altogether, further studies are needed characterizing the intrinsic regulators of hematoma resolution and brain recovery, which could result in the identification of therapeutically feasible molecular targets for ICH.

## Abbreviations

ICH	Intracerebral hemorrhage
SAH	Subarachnoid hemorrhage
MAPK	Mitogen-activated protein kinase
PI3K	Phosphoinositide 3-kinase
BBB	Blood brain barrier
DNA	Deoxyribonucleic acid
NMDA	N-methyl-D-aspartate
MMP-9	Matrix Metalloproteinase 9
AQP-4	Aquaporin-4
AQP-9	Aquaporin-9
PAR	Protease-activated receptor
PAR-1	Protease-activated receptor-1
p38 MAPK	p38 mitogen-activated protein kinase
NLRP3	NACHT, LRR and PYD domains-containing protein 3
PAR-4	Protease-activated receptor-4
RBC	Red blood cell
Hb	Hemoglobin
Hp	Haptoglobin
sCD163	soluble CD163
TLR4	Toll-like receptor-4
IL-1β	Interleukin-1β
TLR-2	Toll-like receptor-2
LRP	Low-density lipoprotein receptor-related protein
HCP1	Heme carrier protein 1
HO	Heme oxygenase
HO-1	Heme oxygenase-1
HO-2	Heme oxygenase-2
CO	Carbon monoxide
Iba1	Ionized calcium binding adaptor molecule 1
GFAP	Glial fibrillary acidic protein
Akt/PKB	Protein kinase B
ASK-1	Apoptotic signaling kinase-1
DMT1	Divalent metal transporter1
FPN-1	Ferroportin-1
Nrf2	Nuclear factor erythroid 2-related factor 2
